# “Not an Either/or Situation”: The Minimization of Violence Against Women In United Kingdom “Domestic Abuse” Policy

**DOI:** 10.1177/1077801220927079

**Published:** 2020-06-22

**Authors:** Jo Aldridge

**Affiliations:** 1 Loughborough University, Leicestershire, UK

**Keywords:** domestic violence and abuse, United Kingdom policy, women’s voices

## Abstract

Proposed new legislation in England and Wales on domestic violence and abuse—the “Domestic Abuse Bill”—is underpinned by changes to criminal law, specifically the introduction of coercive and controlling behavior as set out in the Serious Crime Act 2015. The new Bill commits the British government to four main objectives with, it is claimed, prevention and protection at their heart. What is notable, however, is the rubric shift from “violence” to “abuse” in the proposed new legislation and its subscription to a gender symmetry paradigm that suggests a “watering down” of the government’s response to gendered violence.

## Minimizing “Violence,” Accentuating “Abuse”

This article addresses the introduction of new policy on domestic violence and abuse (DVA) by the British government that shifts the focus away from violence to abuse, and specifically to coercion and control. The new legislation also overlooks the gendered nature of DVA and lacks synergy with both national and international strategies for ending violence against women (VAW). The implications of these changes and oversights are discussed with respect to their impact on the lives of women DVA victims and survivors, to broader gender politics and the “pandemic” of global VAW.

To date there has been no official explanation for the removal of “violence” from the title of the British government’s Domestic Abuse Bill (DAB). It is perhaps representative of a simplistic kind of reductionism or semantic surrogacy whereby “violence” must make way for the a priori language of “abuse,” as if both should not, or cannot be included. This conflicts with many other countries’ policies as well as international strategies and mandates on domestic violence, intimate partner violence and VAW more broadly. In most national and global contexts the preferred term has been “domestic violence and abuse” (a term used throughout this article) or “intimate partner violence” to cover the range of perpetrator behavior in DVA, and the experiences of its victims and survivors. One informal explanation for the removal of “violence” in the United Kingdom legislative context has been that “violence” as a descriptor is not something to which victims/survivors can relate (Aldridge, personal communication, 2018), certainly not as readily as the term “domestic *abuse*”—but without any supportive evidence for this. And yet is also seen by some to be a deliberate move to underplay DVA as a gendered issue.

Although the focus on nonphysical forms of abuse in the proposed new legislation is important as it helps to recognize and cement coercive and controlling behavior in law (note the use by some commentators of “coercive and controlling *violence*”—see Hester et al., 2017), the removal of “violence” as a key rubric suggests a “watering down” or obfuscation of the serious and gendered nature of DVA. In their research with men who use violence in their relationships with women, Kelly and Westmarland (2016) argue that “successive cross-government definitions have disguised, diluted and distorted the reality of men’s violences against women” (p. 114). With respect to the formulation of and consultation on the DAB, this appears to be evident not only in the removal of “violence” from the title of the Bill but also in the British government’s refusal to synergize the proposed new legislation with its Violence Against Women and Girls strategy (VAWG; Home Office, 2016). In 2018, the Home Affairs Committee called for the government to widen the DAB “to be a Violence against Women and Girls and Domestic Abuse Bill” (UK Parliament, 2018), but this did not happen. Following the consultation phase of the Bill the government did concede, however, that it would, “recognise that the majority of victims of abuse are female” in the accompanying statutory guidance as well as “refresh” its VAWG strategy (Home Office, 2019c).

Since 2015, neighboring country Wales has purposefully synchronized legislation on gendered domestic and sexual violence with its DVA policy in its Violence Against Women, Domestic Abuse and Sexual Violence (Wales) Act 2015 (Acts for the National Assembly for Wales, 2015); Ireland retains the rubrical language of violence in its Domestic Violence Act 2018 (Irish Statute Book, 2018), whereas Scotland was the first to refer only to “domestic abuse” in its Domestic Abuse (Scotland) Act 2018 (Acts of the Scottish Parliament, 2018), which came into force in April 2019. In a statement released at the time by the Scottish government, it was stated that the new law represents “groundbreaking legislation that criminalises psychological domestic abuse and coercive and controlling behaviour” and includes “An awareness campaign to increase the public’s understanding of the wide-ranging nature of domestic abuse and to encourage victims of abuse to seek help” (Scottish Government, 2019). It is notable that there is no mention of violence in the statement, and neither is there any reference to the gendered nature of DVA, even though the examples used in the statement are from Women’s Aid and a female DVA survivor.

The lack of reference to violence and the gendered nature of DVA was also notable in the British government’s response to the consultation and draft DAB published in January 2019 (HM Government, 2019). In setting out the government’s four main objectives in the DAB—promoting awareness of DVA, protecting and supporting victims, transforming justice responses, and improving performance in responses to DVA—the Home Secretary Sajid Javid and Justice Secretary David Gauke failed to mention or acknowledge the serious violence women experience at the hands of current or former male partners, or that DVA is a gendered issue. And yet campaigners and women’s rights organizations were unequivocal in their call for the government to recognize the gendered nature of DVA when the Bill was introduced to Parliament in July 2019. As reported in *The Independent* newspaper at the time (Oppenheim, 2019), Adina Claire of national DVA charity Women’s Aid stated,

Women are more likely to have sustained physical or emotional abuse, or violence, which results in serious injury or death. Violence against women is rooted in gender inequality. It is essential that this is explicitly recognised in the domestic abuse bill. To solve any complex social problem, we have to start by defining it, and we know that a gender-neutral definition does not work for this highly gendered issue.

## The Prevalence of VAW in Intimate Relationships

In September 2019, data obtained by the BBC from 43 police forces across the United Kingdom showed that murders as a result of domestic violence were at a 5-year high (BBC, 2019). Furthermore, both national and global evidence on the extent of DVA—perpetrated by males against females in intimate relationships—reveals explicitly its gendered dimensions. Global statistics from the World Health Organization (WHO, 2017), for example, show that 30% of women have experienced some form of physical and/or sexual violence by an intimate partner in their lifetime and that 38% of female murder victims have been killed by a male intimate partner. Evidence from a 2015 European-wide study of VAW, including 42,000 women across 28 member states of the European Union showed,^1^

One in 10 women has experienced some form of sexual violence since the age of 15, and one in 20 has been raped. Just over one in five women has experienced physical and/or sexual violence from either a current or previous partner, and just over one in 10 women indicates that they have experienced some form of sexual violence by an adult before they were 15 years old. (European Union Agency for Fundamental Rights [FRA], 2015, p. 3)

In the United States, findings from the Centers for Disease Control and Prevention (2011) state that the lifetime prevalence of rape by an intimate partner for women was an estimated 8.8%, and that across the United States approximately 15.8% of women experienced other forms of sexual violence by an intimate partner during their lifetime. In addition, women in the United States had a lifetime prevalence of physical violence of 31.5%, this included “being slammed against something” (15.4% of women); just over 13% of women had been hit with a fist or “something hard”; and just under half of United States’ women (47%) had a lifetime prevalence of psychological aggression (at least one act). As U.S. writer Rebecca Solnit (2014) has argued,

Violence is one way to silence people, to deny their voice and their credibility, to assert your right to control over their right to exist. About three women a day are murdered by spouses or ex-spouses in this country. It’s one of the main causes of death for pregnant women in the United States. (p. 6)

Many commentators claim—and this is supported in the evidence cited above—that statistics on the prevalence of DVA across the globe reveal a crisis on a pandemic scale (see Romito, 2008) and that, despite both international and domestic policies and strategies for addressing VAW, including DVA, not enough is being done to address the crisis. The FRA, for example, describes the prevalence of VAW as “an extensive human rights abuse that the EU cannot afford to overlook” (p. 3); the WHO (2017) states VAW—and intimate partner violence and sexual violence, specifically—is “a major public health problem and a violation of women’s human rights.” All of this shows that it would be a serious oversight for governments and states to ignore or overlook the gendered nature of violence in intimate partner relationships and that doing so, in policy, in law and in practice, could have wide-reaching consequences for victims. As Sarah Green (2019), co-director of End Violence Against Women Coalition in the United Kingdom has stated, this would have “troubling ‘real-world consequences’” for women, not least because those professionals such as teachers, social workers and police officers, would be working with a non-gendered definition of DVA that would make it very difficult for them to recognize the signs of and triggers for violence and abuse: “If you preach that it happens to men and women equally, you will not be able to understand the gender stereotyping and sexism element.”

## The Use of Evidence to Promote Gender Symmetry Claims in DVA

Despite this, in the United Kingdom, both policy and practice are being informed by the government’s strategic use of evidence that fails to tell the full story of gendered VAW. Currently, data on DVA in England and Wales are collated through the Crime Survey for England and Wales. In 2017, the published data showed that “an estimated 1.9 million adults aged 16 to 59 years experienced domestic abuse in the last year, according to the year ending March 2017 Crime Survey for England and Wales (1.2 million women, 713,000 men)” (Office for National Statistics, 2017). A significant problem with this finding, however, is that the Crime Survey for England and Wales (CSEW) collects data based on “any incident” of DVA, which could be just one incident, or a series of incidents that are “capped.” The context of the violence or abuse is not taken into account either, hence the reality of the repeated and serious nature of violence and abuse women experience from their male partners in intimate relationships is also overlooked. As Kelly and Westmarland (2016) argue, “Framing domestic violence in terms of incidents—whether in research, policy definitions or practice responses—reflects how violent men describe their behaviour rather than what we know from survivors” (p. 114).

Underlining this argument, in 2016 Walby, Towers, and Francis published results of their analysis of CSEW data over a 10-year period (1994–2014), focusing specifically on “high-frequency victims” (i.e., women victims of DVA) and removing the “cap” on the frequency of victimization (number of crimes committed against them) that surveys, such as the CSEW, include (note, it is not just the CSEW that “caps” repeat crimes, national crime surveys in other countries such as the United States, Canada, and Mexico also do the same). Evidence has shown that capping can reduce the estimates of personal crimes by a third or more, giving a skewed picture of the nature and extent of violent crimes. Following their analysis of 10 years of CSEW data, Walby and colleagues concluded, “Domestic violence (which is disproportionately against women and a gender-saturated category) is often a crime that is repeated by the same perpetrator against the same victim,” and argued further that “capping” as a methodology needed to be both challenged and replaced as it reduced the prevalence of violent crime against women recorded in official statistics. Once the cap is removed, estimates of the extent of violent crime against women, including domestic violence, increase: “In particular the findings demonstrate the significance of the increase in violent crime against those who are repeatedly victimised” (p. 1227).

The British government’s approach to VAW and its refusal to frame this in DVA policy clearly as a gendered issue reinforces Romito’s (2008, p. 43) claims that states and governments across the world continue to deny the reality of male VAW. Romito argues that such tactics are deliberate, “mental operations—ways of seeing, conceptualizing and naming reality—which materialize in behaviour, are deposited as common sense” and that “centre on the interests of those in power.” This is reflected not only in the “skewing” of “knowledge” through flawed methodologies and the misrepresentation of evidence, as has been shown, but also in attempts to equalize DVA perpetration and victimization—to propagate the myth that men and women use violence and abuse equally in intimate relationships. The consequences of this can be, and indeed are, serious for women victims/survivors of DVA. As Kelly and Westmarland (2016) argue, this has led to “many arguing now that domestic violence is not gendered, and thus responses to it should not be” (p. 115). They claim further that this has already had an impact on practice in terms of, “which interventions are deemed appropriate, and who should be prioritised to receive support” (p. 114). And we can already see evidence of this in more recent United Kingdom health practice guidance for practitioners. For example, the Royal College of Nursing’s (2017) most recent guidance on “domestic abuse” for nurses and midwives produced in 2017, starts with the following statistics (putting male victims at the top of the list): “1 in 6 men will experience domestic abuse in their lifetime; 1 in 5 children have been exposed to domestic abuse; 1 in 5 women globally are directly affected by domestic violence.”

## Reinforcing DVA Myths and Women’s Inequalities

A further serious consequence of misrepresenting evidence and producing false or skewed information about DVA (its prevalence and effects) is that it helps to maintain common myths and stereotypes surrounding DVA (see, Haaken, 2010), as well as reinforce negative attitudes toward women victims/survivors, particularly with respect to their help-seeking behavior and fear of not being believed. These issues are illustrated in the following account from DVA survivor, Tula (Aldridge, 2020):

It is very hard when you come up against people who don’t understand what domestic violence is like or think it’s something that men and women experience in the same ways, which is just not true. But ingrained attitudes in people make it harder for women to disclose and seek help. I really found this and it was hard to resist or get around. You just have to fight and keep resisting. You just have to keep going in the knowledge that you know what you went through wasn’t right, that it was abuse, that it was violence, and that you deserve help to escape it, to break free from it. It takes time and it probably takes more time than it should because of people’s attitudes towards domestic violence; but what do they know? Nothing. Only what they’ve been told or read about in the papers, and that’s usually wrong.

One of the outcomes for survivors like Tula is that the inequalities in their lives, that both contribute to and help sustain the public myth and private pain of DVA, are also overlooked as critical indicators in the risk to women of DVA victimization and its perpetuation. Decades of feminist research, politics, and activism have shown us that women’s inequality (economically, politically, their access to services, and so on) also makes them more likely to become trapped in violent and abusive relationships (see, for example, Aldridge, 2013; Nixon & Humphreys, 2010). Underlining this eternal return argument—inequality = victimization = inequality—many commentators argue that it is also “coercive controlling violence” (Hester et al., 2017) in intimate relationships that demonstrates, reinforces, and helps to perpetuate women’s inequality in their own lives and in society more broadly. Indeed, it would be a stretch to consider the evidence on the nature and extent of women’s inequalities in all aspects of their lives and yet subscribe to a gender symmetry paradigm in DVA, that is, to believe that women use violence and abuse equally to men in intimate relationships, rather than feature so frequently as its main and recurring victims. To give some examples, with respect to labor market inequality, evidence from the UN Women (2018) global survey showed that the male employment-to-population ratio was 72.2%, while this was 47.1% for females. Across the world, women are also paid less than men when they are in paid employment; in most countries, women are paid 60-75% of men’s wages. Global evidence also suggests that women spend much less time than men looking after themselves; in almost every country, men spend more hours on leisure time than women do, whereas women are more likely to spend their spare time doing unpaid housework.

In 2017, the Global Gender Gap Report by the World Economic Forum (2017) showed a 32% gender gap for women across four key dimensions: economic participation and opportunity, educational attainment, health, and survival and political empowerment. With respect to both economic participation and political empowerment, the gender gap is only 58% closed and showed a decrease in 2017 and the lowest rate since 2008. The WEC concludes, “The most challenging gender gaps remain in the economic and health spheres. Given the continued widening of the economic gender gap it will now not be closed for another 217 years.”

But it is in criminal justice evidence, and specifically the disproportion in violent offense perpetration, that we can see the most marked difference between men and women. Across the globe, men commit violence at a much higher level than women. In terms of violent offenses including murder, around 80-95% of murders across the globe are committed by men; a global study in 2018 showed that 58% of all female homicides were the result of intimate partner violence (United Nations Office on Drugs and Crime, 2018). In the United Kingdom, men commit 76% of violent offenses and account for 95% of the prison population. The most recent Ministry of Justice (2017) statistics on women and criminal justice in the United Kingdom show that women make up less than 20% of all arrests (and yet, the conviction rate for females was higher at 88%). Given this evidence on the high rate of male-perpetrated criminal offenses by comparison with females—and it is important to remember that while men are much more likely than women to be victims of violence from strangers, the perpetrators here are also much more likely to be male—it is perhaps not surprising that they are also disproportionately responsible for violence and abuse in the context of intimate relationships where the chances of “getting away with it” are much higher; evidence tells us that women experience high numbers of assaults before reporting to the police and many victims never do so (see FRA, 2015), whereas, as Hester et al (2017) show, men tend to “underreport their perpetration of violence and may over report victimization” (p. 417).

## The Continuum of Women’s DVA Experiences

For women survivors of DVA, their experiences of victimization are sometimes difficult to delineate as violence *or* abuse, but it is clear that both have a part to play in women’s chances and opportunities for survival in both the short- and long-term. The shift in focus from violence to abuse in proposed new United Kingdom legislation, coupled with the government’s refusal to couple policy in England with the VAWG strategy, overlooks and denies the violent, destructive *effects* of DVA on women’s lives, regardless of whether the nature of their experiences falls neatly into one or the other category. It also denies women a voice, which is so often critical in ensuring they are believed and can access the necessary help and support to escape and survive. This is supported in Nixon and Humphrey’s (2010) assertion (drawing on Judith Herman’s important work in trauma and recovery) that “individual survivors of domestic violence require a social movement which openly confronts and names the damage and destruction of domestic violence” and that women’s experiences and stories of DVA in all its forms need to be heard in order for them to be listened to and believed rather than “set against a backdrop of . . . minimization and denial” (p. 138).

In recent years, women’s own stories of DVA survival are now more widely heard thanks largely to an upsurge in grassroots feminist activism that has found a wider audience through the internet and new forms of social media. Some of these stories, however,—which, in the United Kingdom have helped to inform and reinforce political agenda setting on DVA—serve to contribute to the “minimization and denial” of the role and (destructive) impact of VAW in intimate relationships, as well as to add further to the semantic confusion in political DVA discourses. A recent example of this, in October 2019, was the reporting of Labor Member of Parliament Rosie Duffield’s personal account of abuse from a former partner, which was described by her House of Commons colleagues and in the press as “moving,” “harrowing,” and “horrifying” (Proctor, 2019). In the *Guardian* news report of Duffield’s account, the subject byline (in red ink) was “domestic violence,” whereas the headline of the piece itself referred only to “domestic abuse” (a similar story on DVA reported by the *Guardian* also in October 2019 demonstrates the same level of semantic confusion and obfuscation; see Usborne, 2019).

While not in any way undermining the seriousness and significance of Duffield’s account, her courage in speaking out, nor the impact on her of the abuse perpetrated by her former male partner, it was interesting to note that she distinguished between her own experiences of “abuse” and popular stereotypes of DVA: “Often we see the same images and stereotypes on T.V. Housing estates, working class families, drunk men coming home from the pub, women surrounded by children and a sequence of shouting followed by immediate physical violence or assault.” Duffield stated, however, that her own experience was different from this stereotype; her partner engaged in coercive control, through “verbal abuse, humiliation and financial control” and this, she said, was interspersed with periods of kindness and romantic gestures; in short: “reward, punishment, promises of happy ever after, alternating with abject rage, menace, silent treatment . . . ”

What Duffield describes here is behavior from her male partner that was erratic but that also escalated in intensity and frequency that eventually led to “rage,” “menace,” and him shouting at her and humiliating her in public. Although clearly a terrible and harrowing experience, as is the case for all women who experience intimate partner violence, what Duffield describes here is mirrored in so many women’s stories of DVA revealed through decades of feminist research. The purpose of her story, however, was to emphasize the difference between physical violence and “domestic abuse” (rather than the scale and complexity of male VAW in intimate relationships) as characterized by coercive control. The effect—certainly in the headline and the start of the report in the *Guardian*—was to obscure or minimize the prevalence and impact of violence in DVA and the fact that DVA reveals a pattern of repetitious and escalatory behavior from male perpetrators that we know makes DVA, or “coercive controlling violence” (Hester et al., 2017), so distinctly a gendered issue. Describing male perpetrator behavior generally in “abusive” relationships by drawing on her own firsthand experience of it, Duffield stated: “They don’t criticize, yell or exert their physical strength in increasingly frightening ways . . . ” but then added, “Not at first,” and went on to say that “domestic violence [*sic*] has many faces.”

What is also interesting about Duffield’s account is that she does not frame “abject rage” or “menace” as violence (rather, as abuse), and her intention to distinguish the “reality” of DVA from the stereotype of the drunken working class man beating his wife does not mean that this picture is not accurate either. Although the role of alcohol misuse in intimate partner violence has been largely, and in many respects purposefully, overlooked in feminist research, a number of studies have demonstrated a link between alcohol misuse and women’s *risk* of DVA that is not intended to detract from or “explain away” male perpetrator behavior and responsibility for VAW. Longitudinal research in Australia, for example, showed that alcohol outlet density “was associated significantly with rates of domestic violence over time” (Livingston, 2011, p. 919), whereas Collins and colleagues’ (2002) research on this subject showed that “the frequency of the husband’s drinking was associated with wife abuse” (p. 388). This is not to suggest that alcohol misuse causes DVA—alcohol misuse prevention does not inevitably equate with the cessation of intimate partner violence—the consumption of alcohol is, however, often used by male perpetrators to excuse their violent behavior toward their partners (see Fazzone et al., 1997).

Despite Duffield’s intentions to distinguish the “stereotypical” TV image of DVA from her own experiences of “domestic abuse,” the familiar trope has some elements of accuracy as does her own story of increasing intimidation and humiliation from her former partner that did not start out that way. Although we know that DVA affects women from all economic and cultural backgrounds across the globe, it is also the case that factors such as lack of economic independence or autonomy, the presence of children in families and women’s need to protect them are critical factors in keeping women trapped in violent and abusive relationships (alongside a range of other complex and interrelated factors). For many women, this situation has only worsened since the economic crash of 2008; in the United Kingdom, for example, this has also occurred as a consequence of more than a decade of austerity that has led to pernicious changes to the benefits system and sweeping cuts to essential DVA services. Research conducted in the United Kingdom by the *Guardian* newspaper in 2018 showed that local council funding for women’s refuges alone had been cut by just under £7 million since 2010 (Grierson, 2018; see also Aldridge, 2013). McRobie (2013, p. 1) argues that collaterally more than 10 years of austerity in the United Kingdom has resulted not only in a lack of support services for women victims—survivors of DVA, but also to an increase in DVA prevalence.

What is becoming increasingly evident, as the austerity measures continue to bite, is how the strain of the recession on women—the disproportionate number of public sector job losses being just one example—has combined with both additional strains that are contributing factors to an increase in domestic violence and, simultaneously, a concerning shrinking of the services available to those experiencing domestic violence.

## Women’s Accounts of Violence and Abuse

Women’s individual stories of violence and abuse in intimate relationships have been critical elements in my own work with women survivors of DVA in both research and practice settings. Many of these women have described their experiences of DVA as living unpredictable, even “schizophrenic” lives, ones which often involved them having to navigate between perpetrator behavior that manifests as violence and abuse one minute and loving kindness the next (but rarely an “either/or” situation). This is reflected in the following account by Katya (Aldridge, 2020). Katya’s partner, Des, rarely used “direct” physical violence, rather she was more likely to experience incidents where she “got in the way” of Des’s outbursts—that manifested as slamming the door on her, punching walls next to her, throwing things around her—but he often also engaged in violent verbal abuse during intense episodes that left her and her young daughter feeling intimidated and afraid, and that had a destructive effect on their lives:

One of the reasons why I found it so hard to make sense of things, to be able to say exactly what it was Des was doing to us, certainly at first, was that there were sometimes long gaps in between his outbursts when he was good and kind and there were no problems. But then it was like he just exploded, like all this pent up frustration and rage burst out and he would just blow. Because he was like this though, because there were long gaps in between, I found it hard to understand or to name his behaviour as violence, as abuse, even though that’s what it was. It happened over time, it was never going to be a one-off incident and when he went off like that—screaming and shouting at me and [my daughter], calling me terrible names, throwing things—I just tried to manage it at the time. But it frightened me and it really scared [my daughter] and I knew in my heart it wasn’t right. It took me a long time though to summon the courage to face it and to name it for what it was—that he was violent and he was an abuser and we were in a one-sided relationship, and that I had to get out.

This type of male perpetrator behavior has been recorded elsewhere in the global research and literature on intimate partner violence, and from countless numbers of women survivors themselves, who often describe the need to negotiate a perilous path between familiarity and uncertainty, between extremes of violence and abuse and benevolence. This kind of unpredictable behavior is also understood as one of the many tactics used by DVA perpetrators to exert control over their victims. Williamson (2010), for example, describes women having to negotiate the “unreality of coercive control” in these kinds of violent and abusive relationships that are defined and sustained by the perpetrator creating an “alien world” to which the woman is forced to become accustomed, but the effects of which are profoundly *destructive* (an essential component in the definition of violence) and traumatic: “While psychologically we negotiate our selves in the wider world everyday, most of us expect to return to a place of safety and calm where we can be ourselves. For women living with domestic violence, this is not possible” (p. 1419).

One of the consequences of failing to delineate and capture the complex nature of violence and abuse in intimate relationships—and for women specifically, of “coercive controlling violence”—in both language and political and public discourse, is that when confronted with DVA people will not know how to respond or help in the most appropriate and effective ways, including women victims themselves, their relatives and friends who want to help them, and professionals working in relevant support services (see Green, 2019). Recognizing and naming DVA are fundamental first steps in women’s help-seeking behavior and in their opportunities to escape violence and abuse (see Seeley & Plunkett, 2002). Evidence tells us that male perpetrators of DVA use a wide range of psychological, emotional, and other practical tactics to deny their partner’s claims of abuse or to minimize and obscure their behavior; this can and does include bringing others into their sphere of manipulation who may not understand the complex nature of DVA or choose not to believe a woman’s claims. Any sense or degree of ambiguity in defining or explicating DVA only serves the perpetrators of DVA while undermining the legitimacy of their partner’s claims and helping to keep others silent on the issue. In his work with DVA perpetrators in the United States, Bancroft (2002) reveals both the myth of others’ “neutrality” in violent and abusive relationships as well as the cost (to women victims) of not speaking out against it:

If you are aware of chronic or severe mistreatment and do not speak out against it, your silence communicates implicitly that you see nothing unacceptable taking place. Abusers interpret silence as approval, or at least as forgiveness. To abused women, meanwhile, the silence means that no one will help—just what her partner wants her to believe. Anyone who chooses to quietly look the other way therefore unwittingly becomes the abuser’s ally. (p. 287)

The minimization and subjugation of violence in favor of the more generic language of “abuse,” as is evident in new United Kingdom DVA policy, for example, could serve to obscure or diminish understanding about what constitutes DVA and thus women’s chances of escaping it. While recognizing and including coercion and control in DVA policy and practice, as well as in political discourse is clearly important, this should not mean overlooking, minimizing, or obfuscating violence in its destructive definitional sense (whether physical, sexual, verbal, or emotional), nor in terms of its devastating physical, psychological, and/or emotional effects on women who experience it. As has been stated, too often this is not an “either/or” situation for women; this is illustrated in Katya’s further description of Des’s behavior:

He didn’t even have to say or do anything, sometimes he just looked at me with such loathing, with such intense hatred, like he wanted to kill me and it was so powerful that it had a traumatic effect on me emotionally and physically; it made me think there was something wrong with me and that he would carry it through. It terrified me so much I just didn’t know what to do.

## Women and Children First

Research conducted for the United Kingdom Home Office published in 2019 (Home Office, 2019b) showed the economic and social costs of DVA (year ending March 31, 2017) to be £66 billion, with the biggest proportion of these costs being the “physical and emotional harms incurred by victims (£47bn)” (p. 1). This fact alone should incentivize any government to take strategic action over DVA. In response, the British government’s objectives in addressing DVA—strategies set out in the DAB—include greater focus on the safety of victims, and transforming the justice process. The latter is not an inconsiderable task given the sweeping cuts to essential services that have occurred as a result of more than 10 years of austerity measures (see, for example, Aldridge, 2013) in the United Kingdom, as well as the legacy of police and criminal justice failings in response to DVA incidents and cases (see Her Majesty’s Inspectorate of Constabulary, 2014). Nevertheless, the government is committed to the latter through, it is claimed, greater focus on victims’ safety in both the criminal and family courts, as well as through a review of the perpetrator journey “from identification to rehabilitation” (HM Government, 2019).

While putting the spotlight (or at least some of it) on rehabilitation is important, evidence on the success of perpetrator programs is largely contested. “Some evaluations have concluded they do reduce violence, whereas others claim they do not and may even make things worse” (Westmarland et al., 2010, p. 1; see also Bancroft, 2002). Furthermore, what this commitment to a review of the perpetrator’s “journey” ignores is that women continue to be held responsible for their own safety, and particularly that of their children, in DVA contexts (and many are forced to give them up to the care system as a consequence) regardless of the progress or journey male perpetrators make on the road to “recovery.” It also fails to acknowledge the importance of prevention through education and the need to address women’s inequality as strategic ways in which DVA can and should be addressed. With respect to better protecting women victims of DVA and their children, some local authorities in the United Kingdom have had success with the adoption of the Safe and Together child welfare model (Safe and Together Institute, 2019), based on David Mandel’s program in the United States that prioritizes keeping children safe with their non-offending parent. The model also puts the focus on the perpetrator as fully responsible for violence and abuse in family settings. But it is perpetrator *attitudes and responses* to women (and children) that are significant stumbling blocks to prevention, especially as evidence tells us that many of the kinds of entrenched attitudes male perpetrators demonstrate are inevitably linked to an inherent chauvinism and belief in women’s fundamental inequality and subservience.

It is difficult to imagine how the transformation of justice and closer focus on perpetrator rehabilitation—key objectives in the British government’s new DVA agenda—will have much or any long-lasting effect without serious attention to women’s domestic and broader inequalities. Following years of counseling male perpetrators of DVA in the United States, Bancroft (2002) proposes that ingrained ideas and beliefs about women and their place in society “make it difficult for them to imagine being in a respectful and equal relationship with a woman” (p. xxi). Although this might offer some hope for change (e.g., in male attitudes and beliefs), *how* to effect such transformation rests on setting and achieving much more exigent goals that include, as Bancroft proposes, furthering both individual and community understanding about how abusive thinking works as well as its origins. “The sources are many,” he argues:

The most important ones include the family he grows up in, his neighborhood, the television he watches and books he reads, jokes he hears, messages that he receives from the toys he is given, and his most influential adult role models. In short, “from the full range of his experiences within his *culture*.” (p. 319)

## Conclusion

It is true that DVA occurs in same-sex relationships; men also experience violence and abuse in intimate relationships, although they do so far less frequently than women and are also significantly more likely to be the perpetrators of DVA. Evidence also tells us that women use violence in very different ways from men in these contexts. As Hester (2012) states,

Research from a range of methodologies not only indicates that both women and men can be violent but also highlight gender differences in the extent, severity, and impact of [intimate partner violence], with women less likely to use the ongoing pattern of “battering” involving “coercive controlling tactics along with systematic threats and use of violence.” (p. 2)

It is also the serious repetitious and escalatory nature of male VAW in intimate relationships that points to the gendered nature of DVA. DVA statistics for the United Kingdom show that fatal violence in intimate relationships is increasing and that across the globe almost one third (30%) of women have experienced physical and/or sexual violence from a male partner (WHO, 2017). All evidence suggests that not enough is being done practically and in law to protect women from DVA. The new legislation in the United Kingdom promises a raft of provisions, including improved protection and support for victims and greater focus on bringing perpetrators to justice, as well as to, “raise awareness about the devastating impact of domestic abuse on victims and their families” (Home Office, 2019a). Nevertheless, without greater recognition of violence and abuse as gendered issues in intimate relationships, and the necessary synergizing with the government’s VAWG strategy—and by extension with international strategies to eliminate gendered violence (United Nations Human Rights Office of the High Commissioner, 1993)—it is unlikely the lives of women victims are going to improve, including their chances of escape and survival. It is also difficult to understand how a more strategic focus on raising awareness about DVA—as set out in the government’s new “domestic abuse” policy—is easily reconciled with an approach that appears to minimize violence in both in semantic terms and in its application.

One of the consequences of an evident heterogeneity in DVA policy and discourse is to muddy the definitional waters and to polarize understanding between public perception of DVA and the private pain of those who experience it. In describing VAW in intimate relationships simply as “abuse” (as a cover-all for all types of DVA situations), in denying equal weight to violence in all its destructive forms, the effect is both a dilution and detraction from the serious, often fatal consequences of violence and abuse in intimate relationships for women and to overlook the gendered nature of “coercive and controlling violence.” It also serves to minimize, and at the same time lend equivalence to, what is a complex and gendered social problem. With respect to the latter, in the move to recognize and prioritise coercive control, particularly in new United Kingdom legislation, what has been overlooked is the fact that it is this aspect of DVA perpetration specifically that delineates its gendered nature (see Hester et al., 2017).

To some extent this oversight can be seen as symptomatic of a much wider tactic of resistance and refutation in patriarchal systems that helps to maintain a catalog of public myths about DVA (see Haaken, 2010), that in turn have helped to influence government and state policies and practices, as well as negatively affect the lives of women victims-survivors across the globe. It was nearly two decades ago that Heise and colleagues stated (2002),

Despite its high costs, almost every society in the world has social institutions that legitimize, obscure and deny abuse. The same acts that would be punished if directed at an employer, a neighbor, or an acquaintance often go unchallenged when men direct them at women, especially within the family. (p. 5)

Considered from a United Kingdom perspective, it is disheartening to realize how little has changed in the intervening years.

## Notes

1. This article was written and submitted for publication before the onset of the global pandemic crisis.

## References

[bibr1-1077801220927079] Acts for the National Assembly for Wales. (2015). Violence against women, domestic abuse and sexual violence (Wales) Act 2015. https://www.legislation.gov.uk/anaw/2015/3/contents

[bibr2-1077801220927079] Acts of the Scottish Parliament. (2018). Domestic Abuse (Scotland) Act 2018. http://www.legislation.gov.uk/asp/2018/5/contents/enacted

[bibr3-1077801220927079] Aldridge J. (2013). Identifying the barriers to women’s agency in domestic violence: The tensions between women’s personal experiences and systemic responses. Social Inclusion, 1(1), 1–17.

[bibr4-1077801220927079] Aldridge J. (2018). Personal communication and discussion at national Domestic Violence and Abuse Forum meeting (involving government representatives and other DVA organizsations).

[bibr5-1077801220927079] Aldridge J. (2020). The survivor testimonies of women victims and survivors of domestic violence and abuse. Loughborough University.

[bibr6-1077801220927079] Bancroft L. (2002). “Why does he do that?” Inside the minds of angry and controlling men. Penguin Group.

[bibr7-1077801220927079] BBC. (2019). Domestic violence killings reach five-year high: https://www.bbc.co.uk/news/uk-49459674

[bibr8-1077801220927079] Centers for Disease Control and Prevention. (2011). Prevalence and characteristics of sexual violence, stalking and intimate partner violence victimization: National intimate partner and sexual violence survey. https://www.cdc.gov/mmwr/preview/mmwrhtml/ss6308a1.htm?s_cid=ss6308a1_e

[bibr9-1077801220927079] Collins J. J. Kroutil L. A. Roland E. J. Moore-Gurrera M. (2002). Issues in the linkage of alcohol and domestic violence services. In Gallanter M. (Ed.), Recent developments in alcoholism (pp. 387–405). Springer. 10.1007/0-306-47141-8_20 9122503

[bibr10-1077801220927079] European Union Agency for Fundamental Rights. (2015). Violence against women: An EU-wide survey. Main results. https://fra.europa.eu/sites/default/files/fra_uploads/fra-2014-vaw-survey-main-results-apr14_en.pdf

[bibr11-1077801220927079] Fazzone P. A. Holton J. K. Reed B. G. (1997). Substance abuse treatment and domestic violence. Substance Abuse and Mental Health Services Administration. 22514831

[bibr12-1077801220927079] Green S. (2019, July 16). Domestic abuse bill condemned for ignoring “gendered nature” of violence amid austerity cuts. The Independent. https://www.independent.co.uk/news/uk/home-news/domestic-abuse-bill-parliament-criticism-theresa-may-women-men-violence-a9007151.html

[bibr13-1077801220927079] Grierson J. (2018, March 23). Council funding for women’s refuges cut by nearly £7m since 2010. The Guardian. https://www.theguardian.com/society/2018/mar/23/council-funding-womens-refuges-cut-since-2010-england-wales-scotland

[bibr14-1077801220927079] Haaken J. (2010). Hard knocks: Domestic violence and the psychology of storytelling. Routledge.

[bibr15-1077801220927079] Heise L. Ellsberg M. Gottmoeller M. (2002). A global overview of gender-based violence. International Journal of Gynecology & Obstetrics, 78, 5–14. 10.1016/S0020-7292(02)00038-3 12429433

[bibr16-1077801220927079] Her Majesty’s Inspectorate of Constabulary. (2014). Everyone’s business: Improving the police response to domestic abuse. https://www.justiceinspectorates.gov.uk/hmicfrs/wp-content/uploads/2014/04/improving-the-police-response-to-domestic-abuse.pdf

[bibr17-1077801220927079] Hester M. (2012). Portrayal of women as intimate partner domestic violence perpetrators. Violence Against Women, 18(9), 1067–1082. 22996629 10.1177/1077801212461428

[bibr18-1077801220927079] Hester M. Jones C. Williamson E. Fahmy E. Feder G. (2017). Is it coercive controlling violence? A cross-sectional domestic violence and abuse survey of men attending general practice in England. Psychology of Violence, 7(3), 417–427.

[bibr19-1077801220927079] HM Government. (2019, January). Transforming the response to domestic abuse: Consultation response and draft Bill. https://assets.publishing.service.gov.uk/government/uploads/system/uploads/attachment_data/file/772202/CCS1218158068-Web_Accessible.pdf

[bibr20-1077801220927079] Home Office. (2016). Strategy to end violence against women and girls: 2016-2020. https://www.gov.uk/government/publications/strategy-to-end-violence-against-women-and-girls-2016-to-2020

[bibr21-1077801220927079] Home Office. (2019a). Domestic abuse bill: Overarching fact sheet. https://www.gov.uk/government/publications/domestic-abuse-bill-2019-factsheets/domestic-abuse-bill-2019-overarching-fact-sheet

[bibr22-1077801220927079] Home Office. (2019b). The economic and social costs of domestic abuse. https://assets.publishing.service.gov.uk/government/uploads/system/uploads/attachment_data/file/772180/horr107.pdf

[bibr23-1077801220927079] Home Office. (2019c). Policy paper: Statutory definition of domestic abuse fact sheet. https://www.gov.uk/government/publications/domestic-abuse-bill-2019-factsheets/statutory-definition-of-domestic-abuse-fact-sheet

[bibr24-1077801220927079] Irish Statute Book. (2018). Domestic violence Act 2018. http://www.irishstatutebook.ie/eli/2018/act/6/enacted/en/html

[bibr25-1077801220927079] Kelly L. Westmarland N. (2016). Naming and defining “domestic violence”: Lessons from research with violent men. Feminist Review, 112, 113–127.

[bibr26-1077801220927079] Livingston M. (2011). A longitudinal analysis of alcohol outlet density and domestic violence. Addiction, 106(5), 919–925. 21205052 10.1111/j.1360-0443.2010.03333.x

[bibr27-1077801220927079] McRobie H. (2013). Austerity and domestic violence: Mapping the damage. Open Democracy. https://www.opendemocracy.net/en/5050/austerity-and-domestic-violence-mapping-damage/

[bibr28-1077801220927079] Ministry of Justice. (2017). Statistics on women and the criminal justice system 2017. https://assets.publishing.service.gov.uk/government/uploads/system/uploads/attachment_data/file/759770/women-criminal-justice-system-2017.pdf

[bibr29-1077801220927079] Nixon J. Humphreys C. (2010). Marshalling the evidence: Using intersectionality in the domestic violence frame. Social Politics, 17(2), 137–158.

[bibr30-1077801220927079] Office for National Statistics. (2017). Domestic abuse in England and Wales: Year ending March 2017. https://www.ons.gov.uk/peoplepopulationandcommunity/crimeandjustice/bulletins/domesticabuseinenglandandwales/yearendingmarch2017

[bibr31-1077801220927079] Oppenheim M. (2019, July 16). Domestic abuse bill condemned for ignoring “gendered nature” of violence amid austerity cuts. The Independent. https://www.independent.co.uk/news/uk/home-news/domestic-abuse-bill-parliament-criticism-theresa-may-women-men-violence-a9007151.html

[bibr32-1077801220927079] Proctor K. (2019, October 2). Labour MP moves colleagues to tears with domestic abuse story. https://www.theguardian.com/society/2019/oct/02/labour-mp-moves-colleagues-to-tears-with-domestic-abuse-story

[bibr33-1077801220927079] Romito P. (2008). A deafening silence: Hidden violence against women and children. Policy Press.

[bibr34-1077801220927079] Royal College of Nursing. (2017). Domestic abuse: RCN guide for nurses and midwives to support those affected by domestic abuse.

[bibr35-1077801220927079] Safe and Together Institute. (2019). https://safeandtogetherinstitute.com

[bibr36-1077801220927079] Scottish Government. (2019). Domestic Abuse Act in force. https://www.gov.scot/news/domestic-abuse-act-in-force/

[bibr37-1077801220927079] Seeley J. Plunkett C. (2002). Women and domestic violence: Standards for counselling practice. Salvation Army Crisis Services.

[bibr38-1077801220927079] Solnit R. (2014). Men explain things to me, and other essays. Granta.

[bibr39-1077801220927079] UK Parliament. (2018). Domestic abuse: Summary. https://publications.parliament.uk/pa/cm201719/cmselect/cmhaff/1015/101503.htm

[bibr40-1077801220927079] United Nations Human Rights Office of the High Commissioner. (1993). Declaration on the elimination of violence against women. https://www.ohchr.org/EN/ProfessionalInterest/Pages/ViolenceAgainstWomen.aspx

[bibr41-1077801220927079] United Nations Office on Drugs and Crime. (2018). Global study on homicide: Gender-related killing of women and girls. https://www.unodc.org/documents/data-and-analysis/GSH2018/GSH18_Gender-related_killing_of_women_and_girls.pdf

[bibr42-1077801220927079] UN Women. (2018). Facts and figures: Economic empowerment. http://www.unwomen.org/en/what-we-do/economic-empowerment/facts-and-figures

[bibr43-1077801220927079] Usborne S. (2019, October 10). Domestic murderers are often likeable men—That’s how they have been able to abuse women. The Guardian. https://www.theguardian.com/lifeandstyle/2019/oct/10/domestic-murderers-are-often-likable-men-thats-how-they-have-been-able-to-abuse-women

[bibr44-1077801220927079] Walby S. Towers J. Francis B. (2016). Is violent crime Increasing or decreasing? A new methodology to measure repeat attacks making visible the significance of gender and domestic relations. British Journal of Criminology, 56(6), 1203–1234.

[bibr45-1077801220927079] Westmarland N. Kelly L. Chalder-Mills J. (2010). Domestic violence perpetrator programmes: What counts as success? http://dro.dur.ac.uk/11515/1/11515.pdf?DDD34+dss4ae

[bibr46-1077801220927079] Williamson E. (2010). Living in the world of the domestic violence perpetrator: Negotiating the unreality of coercive control. Violence Against Women, 16(12), 1412–1423. 21164217 10.1177/1077801210389162

[bibr47-1077801220927079] World Economic Forum. (2017). Global gender pay gap report. United Nations.

[bibr48-1077801220927079] World Health Organization. (2017). Violence against women: Key facts. http://www.who.int/news-room/fact-sheets/detail/violence-against-women

